# Donor-derived cell-free DNA detects kidney transplant rejection during nivolumab treatment

**DOI:** 10.1186/s40425-019-0653-6

**Published:** 2019-07-12

**Authors:** Daan P. Hurkmans, Jeroen G. H. P. Verhoeven, Kitty de Leur, Karin Boer, Arjen Joosse, Carla C. Baan, Jan H. von der Thüsen, Ron H. N. van Schaik, Ron H. J. Mathijssen, Astrid A. M. van der Veldt, Dennis A. Hesselink

**Affiliations:** 1 Department of Medical Oncology, Erasmus MC Cancer Institute, University Medical Center Rotterdam, Rotterdam, The Netherlands; 2 Department of Internal Medicine, Division of Nephrology and Transplantation, University Medical Center Rotterdam, Rotterdam, The Netherlands; 3 Rotterdam Transplant Group, University Medical Center Rotterdam, Rotterdam, The Netherlands; 4 Department of Pathology, University Medical Center Rotterdam, Rotterdam, The Netherlands; 5 Department of Clinical Chemistry, University Medical Center Rotterdam, Rotterdam, The Netherlands; 6 Department of Radiology & Nuclear Medicine, Erasmus MC, University Medical Center Rotterdam, Rotterdam, The Netherlands

**Keywords:** Allograft rejection, Anti-PD-1, Dd-cfDNA, Melanoma, Donor-derived cell-free DNA, Immune checkpoint inhibition, Immunotherapy, Nivolumab, Solid organ transplantation, Kidney transplantation

## Abstract

**Background:**

In solid organ transplant (SOT) recipients, transplant rejection during immune checkpoint inhibitor (ICI) treatment for cancer is a clinical problem. Donor-derived cell-free DNA (dd-cfDNA) can be detected in blood and is a sensitive biomarker for diagnosis of acute rejection in SOT recipients. To our best knowledge, this is the first case report of a kidney transplant recipient with advanced cancer treated with ICI who was monitored with dd-cfDNA.

**Case presentation:**

A 72-year old female with a long-standing renal transplant was diagnosed with advanced melanoma in 2018 and was treated with the anti-PD1 antibody nivolumab. Within 12 days after the first administration of nivolumab, dd-cfDNA ratio increased to 23%, suggesting allograft rejection. Her kidney transplant function deteriorated and acute rejection was confirmed by renal transplant biopsy. As the rejection could not be controlled despite immunosuppressive treatment, a transplant nephrectomy was necessary and haemodialysis was started. Immunological analysis of the renal explant showed infiltration of alloreactive, nivolumab-saturated, PD1+ cytotoxic T cells. After transplant nephrectomy, she experienced nivolumab-related toxicity and rapid disease progression.

**Conclusion:**

Clinicians prescribing ICIs should be aware that SOT recipients are at risk of transplant rejection as a result of T cell activation. Dd-cfDNA is a sensitive biomarker and should be further studied for early detection of transplant rejection. Immunological analysis of the kidney explant showed marked graft infiltration with alloreactive PD-1^+^ cytotoxic T cells that were saturated with nivolumab.

## Background

Immune checkpoint inhibitors (ICIs) have significantly improved the overall survival of patients with advanced malignancies, including advanced stage melanoma [1]. The monoclonal antibody nivolumab blocks the inhibitory immune checkpoint receptor programmed death-1 (PD-1), thereby promoting the anti-tumor immune response [2]. This is particularly hazardous for solid organ transplant (SOT) recipients who may develop acute rejection as a result of enhanced T cell activation [3]. As SOT recipients have an increased risk to develop ICI-responsive malignancies, including melanoma and cutaneous squamous cell carcinoma [4, 5], ICI-induced SOT rejection is a clinical problem. For adequate patient counselling and early intervention during ICI treatment, biomarkers for early detection of acute rejection are needed. However, conventional biomarkers to monitor SOT integrity have a low sensitivity and specificity [6].

Donor-derived cell-free DNA (dd-cfDNA) can be detected in blood and urine of SOT recipients and has been shown to be a potentially useful biomarker for the early diagnosis of acute rejection of kidney transplants [7]. In kidney transplant recipients, dd-cfDNA levels of < 1% of total cfDNA appear to reflect the absence of active rejection whereas levels > 1% seem to indicate active rejection [7]. However, many questions regarding the clinical utility of dd-cfDNA monitoring following SOT remain and this is not standard practice (reviewed in Verhoeven et al.) [7]. Here, a kidney transplant recipient is described who experienced severe acute allograft rejection during ICI therapy for metastatic melanoma. In the current analysis, dd-cfDNA was evaluated as a potential sensitive biomarker for detection of transplant rejection in a cancer patient treated with ICIs. Second, to understand the pathophysiology of this ICI-induced rejection, graft-infiltrating leucocytes were isolated and characterized.

## Case presentation

In 2018, a 72-year-old female with a long-standing renal transplant was diagnosed with metastatic BRAF-wildtype melanoma, 5 years after a cutaneous melanoma (Breslow thickness 0.8 mm) had been radically excised. She presented with a solitary large left axillary metastasis of 6 cm which encased the axillary artery and the plexus brachialis, resulting in edema and paralysis of her left arm. The patient had received a deceased donor kidney transplant in 2013 due to end-stage renal disease caused by hypertensive nephropathy and a unilateral nephrectomy because of renal cell carcinoma (T2N0M0) in 2006. Apart from the development of post-transplantation diabetes mellitus, the clinical course after her transplantation had been uneventful. At the time of melanoma diagnosis, she had a stable renal function with limited proteinuria (urinary protein to creatinine ratio of 33 g/mol) and a serum creatinine concentration of 150 umol/L, corresponding to an eGFR of 30 mL/min per 1.73 m^2^ (CKD-EPI formula) [8].

The large left axillary mass was considered unresectable. After radiotherapy combined with hyperthermia, she had progressive disease with pulmonary and distant lymph node metastases. She was carefully counselled about ICI-associated side effects, specifically about the possibility of renal allograft rejection. Progressive axillary metastasis with severe vascular and neurologic complications led to the shared decision to start first-line nivolumab (3 mg/kg Q2W). The immunosuppressive regimen consisting of tacrolimus (1.5 mg q.d.) and mycophenolate mofetil (500 mg b.i.d.) was switched to prednisolone (20 mg q.d.) and nivolumab was administered 1 week thereafter.

Twelve days after first nivolumab administration, the patient presented with nausea, vomiting, loose stools and abdominal pain located at the site of her transplant. Laboratory investigation demonstrated severe renal insufficiency with a serum creatinine of 549 umol/L. A kidney transplant biopsy was performed and demonstrated extensive acute ischemic changes with capillary endothelial necrosis, tubular epithelial degeneration, edema and haemorrhage, consistent with infarction (Fig. 3a**)**. These findings were interpreted as acute kidney transplant rejection and methylprednisolone pulse therapy (1000 mg intravenously for 3 consecutive days) and haemodialysis were initiated. Because of ongoing rejection despite methylprednisolone treatment, prednisolone was discontinued and transplant nephrectomy was performed. Because of advanced malignancy, T lymphocyte-depleting antibodies were not administered.

**Fig. 1:**
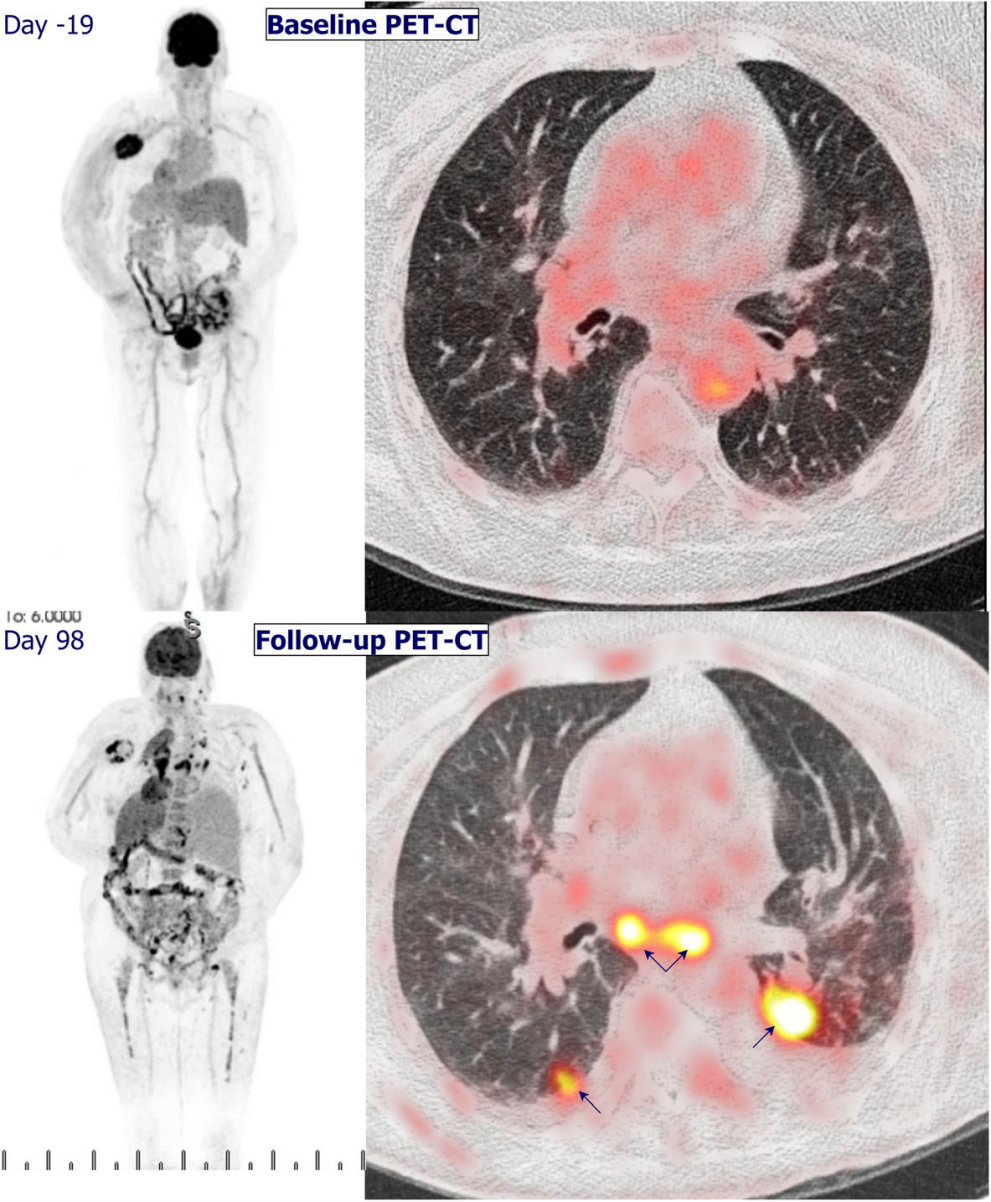
^18^F-FDG PET-CT revealed progressive disease at three months after start of nivolumab, with new lung and lymph node metastases. Pleural effusion was present

After transplant nephrectomy, nivolumab was continued for a period of 8 weeks. As she experienced immune-related adverse events, including pneumonitis grade 2 and colonoscopy-conformed colitis grade 2 (common terminology criteria for adverse events version 4.03), nivolumab was discontinued and prednisolone was initiated. Three months after the start of nivolumab, ^18^F-FDG PET-CT revealed progressive disease with new lung and lymph node metastases (Fig. 1). The patient decided to stop haemodialysis and died 5 months after the start of nivolumab.

### Dd-cfDNA reveals acute allograft rejection

**Fig. 2:**
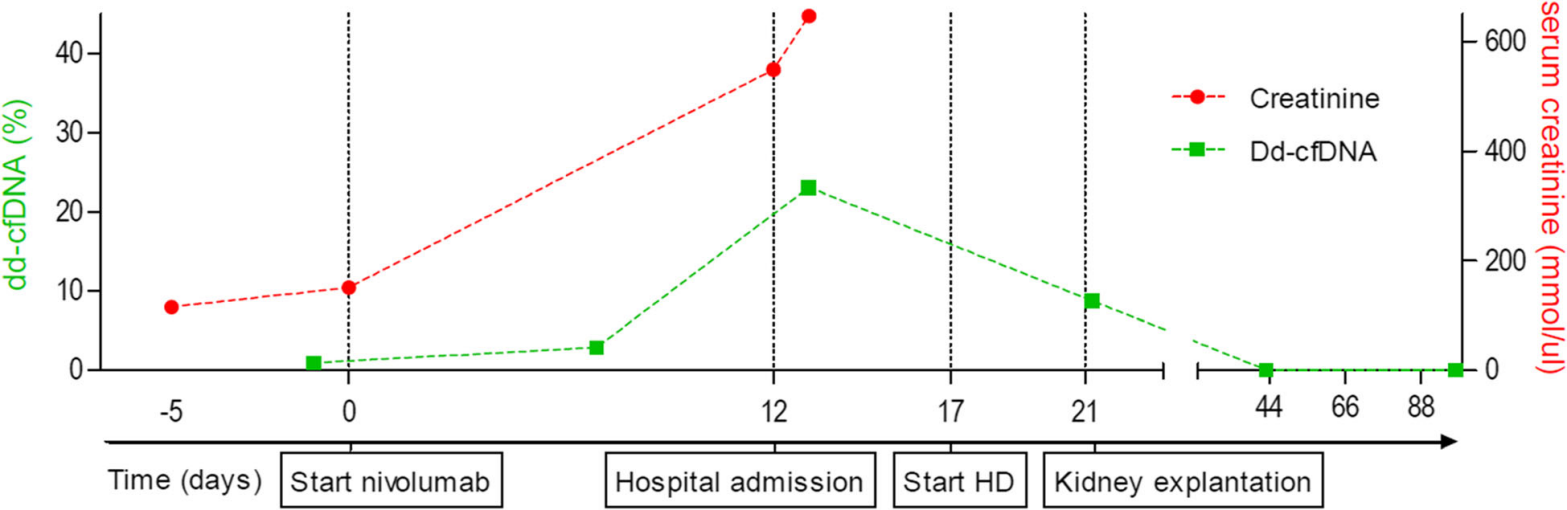
Time course of the percentage plasma dd-cfDNA (in green) and serum creatinine concentration (in red), in relation to important clinical events. During the hospital admission, hemodialysis (HD; day 17) was initiated. Dd-cfDNA levels declined from 23 to 8.8% 3–5 h after kidney explantation. Dotted lines are added to connect separate measurements of creatinine and dd-cfDNA. Of note, no comparative serum creatinine measurement was performed at 7 days after the first administration of nivolumab

The patient participated in the MULTOMAB clinical trial, (see Dutch Trial Register number NTR7015), in which blood is collected prospectively for translational purposes. After kidney transplant rejection, previously obtained blood samples were analyzed for dd-cfDNA. Dd-cfDNA was expressed as a percentage of total cfDNA (see Methods section below). Prior to administration of nivolumab, dd-cfDNA was low (0.9%; Fig. 2). One week after administration of nivolumab, dd-cfDNA increased to 2.9%, indicating active rejection of the allograft. At the time of rejection, 12 days after first administration of nivolumab, dd-cfDNA increased to a maximum of 23.1%. Dd-cfDNA levels declined to 8.8, 0.1 and 0.0% at 3–5 h, 22 days and 77 days after transplant explantation, respectively, corresponding with the half-life of dd-cfDNA [9].

### Acute vascular rejection with viable graft infiltrating lymphocytes

**Fig. 3:**
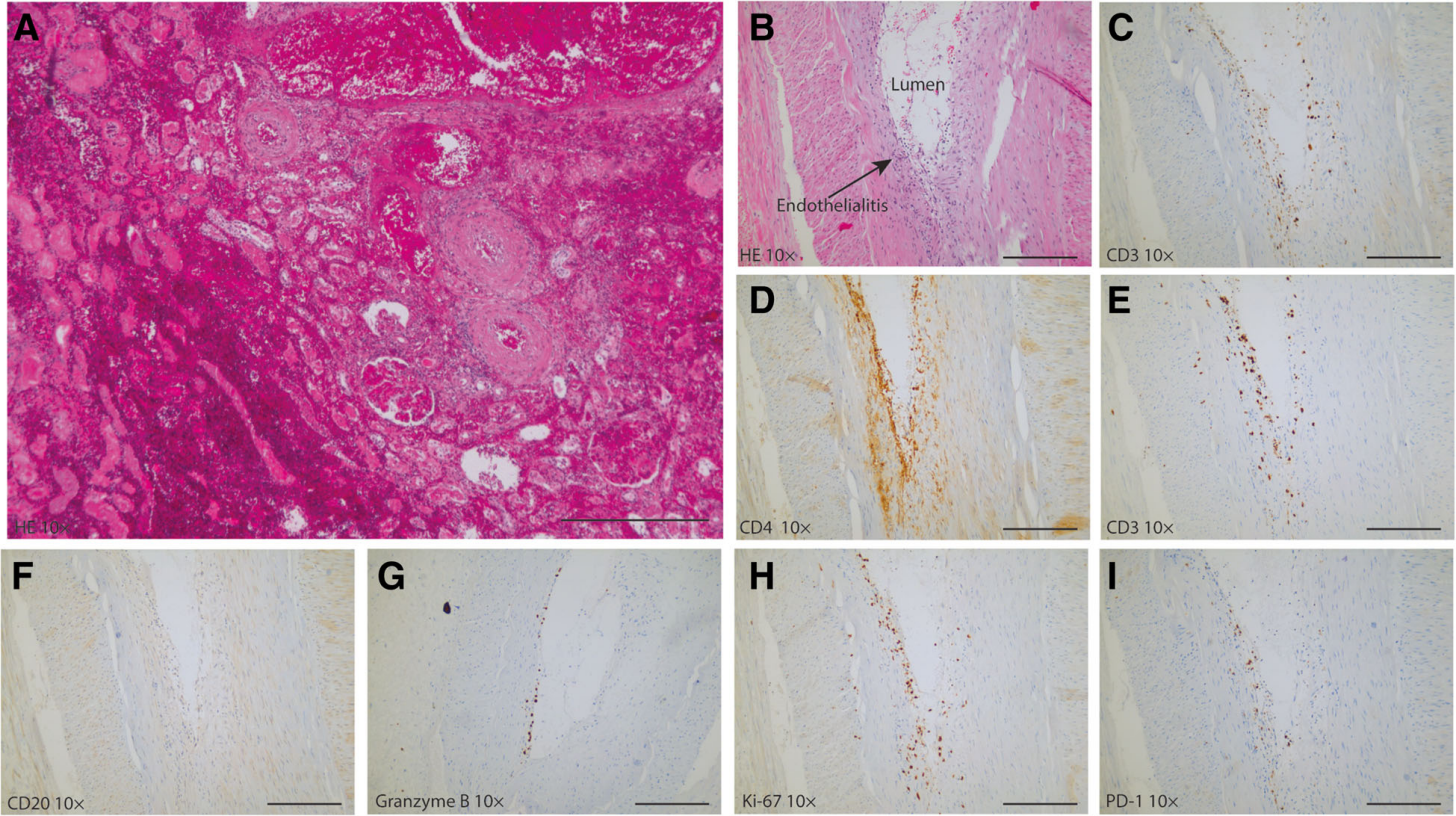
Histology of the renal graft at the time of the kidney transplant biopsy and the explantation under nivolumab treatment (250 μm scale bar). Immunohistochemistry of the explanted kidney. **a** HE staining of the kidney biopsy shows diffuse cortical necrosis, hemorrhage and glomerular congestion. **b** HE staining of the renal explant shows moderate endothelialitis with focal fibrin deposition. **c**-**i** immunohistochemistry of the explanted kidney. **c**-**e** CD3^+^, CD4^+^ and CD8^+^ T-cells are present. **f** no CD20^+^ B-cells are present. **g** and **h** indicates the presence granzyme producing cells and proliferating cells (Ki-67) cells. Overall, there is influx of PD-1^+^ granzyme B-producing CD8^+^ T-cells in the vascular wall with endothelialitis. Magnification: 10x

Histopathological examination of the explanted kidney allograft demonstrated severe vascular, acute T-cell mediated rejection with an almost entirely necrotic kidney parenchyma with hemorrhage and moderate endothelialitis with focal fibrin deposition (Fig. 3b). CD3^+^ T lymphocytes were found subendothelially (Fig. 3c) and included both CD4^+^ (Fig. 3d) and CD8^+^ T cells (Fig. 3e). No CD20^+^ B lymphocytes were identified (Fig. 3f). The cytotoxic CD8^+^ T cells were active and viable, as evidenced by the presence of intracellular granzyme B (Fig. 3g) and Ki-67 (Fig. 3h), reflecting their cytotoxic potential and proliferation, respectively. PD-1^+^ staining was also seen in the vessel wall (Fig. 3i).

**Fig. 4:**
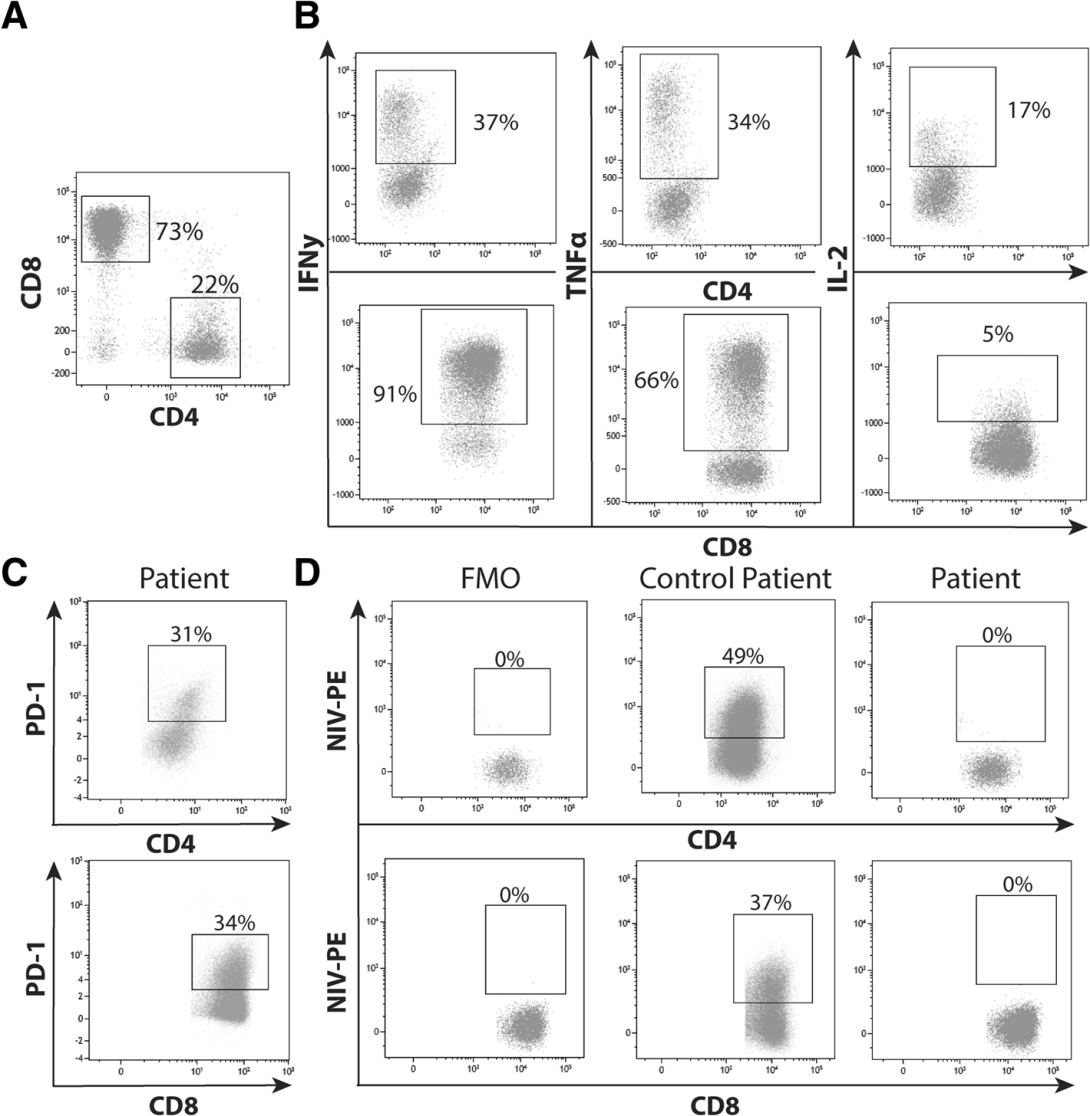
Phenotyping of the graft infiltrating lymphocytes isolated from the explanted kidney during nivolumab treatment. **a** Unstimulated graft infiltrating-lymphocytes were gated by size and granularity in the forward and side scatter. CD4^+^ and CD8^+^ T cells were gated within total CD3^+^ cells. **b** Intracellular IFN-γ, TNFα and IL-2 was determined in CD3^+^CD8^−^ (representing the CD4^+^ population) and CD8^+^ T cells at 3 h of stimulation with PMA/ionomycin. **c** Presence of the presence of PD-1 in CD4^+^ and CD8^+^ T-cells was also determined. **d** Blockade of the PD-1 receptor by nivolumab was demonstrated by adding conjugated nivolumab to these cells and was compared with graft infiltrating lymphocytes of a rejected kidney from a patient who was not treated with nivolumab

Despite the necrotic status of the renal explant, viable lymphocytes were revealed, which mainly consisted of CD3^+^ T cells (59%). Within the total CD3^+^ T cell population, the CD4^+^:CD8^+^ ratio was approximately 1:3 (22% CD4^+^ and 73% CD8^+^, Fig. 4a). Cytokines, such as IFN-y, TNF and IL-2, play an important role in the immune response that mediate allograft rejection. The amount of these pro-inflammatory cytokines, produced by T-cells, indicates whether these cells are activated. After polyclonal stimulation, the capacity of the T cells to produce IFN-γ, TNFα and IL-2 was measured [10]. CD8^+^ T cells had a higher capacity than CD4^+^ T cells to produce IFN-γ (91% vs. 37%; Fig. 4b) and TNFα (66% vs. 34%), whereas CD4^+^ T cells showed a higher capacity for IL-2 production (5% vs. 17%).

### Nivolumab PD-1 occupancy on graft infiltrating lymphocytes

Further immunological analysis was performed to examine whether nivolumab was successfully bound to the graft infiltrating lymphocytes (GILs), which were considered to have caused rejection. Among the GILs, PD-1 was expressed on both the CD4^+^ and CD8^+^ T cells (31 and 34%, respectively; Fig. 4c), indicating that the receptor for nivolumab was present on the surface of these cells. To determine the amount of free PD-1 binding places on the GILs in the explant, conjugated nivolumab was added to the explant of both the current and a control patient, who experienced an acute rejection without ICI. In the renal explant of the control patient, nivolumab binding capacity was 49% of CD4^+^ and 37% CD8^+^ T-cells (Fig. 4d), whereas conjugated nivolumab was not able to bind CD4^+^ and CD8^+^ T-cells (0 and 0%, respectively) in the nivolumab-treated patient.

## Discussion and conclusion

Here a melanoma patient with a kidney transplant is reported who developed a fulminant acute kidney allograft rejection 2 weeks after the start of nivolumab treatment. Dd-cfDNA was measured in this cancer patient to monitor allograft integrity and detect potential allograft rejection at an early stage during treatment with an ICI. Previously, it has been reported that quantification of so called dd-cfDNA can be useful to detect allograft rejection. Cell-free DNA is degraded into non-encapsulated DNA and released after cell death, or by active secretion of cells. During SOT rejection, the cells of donor origin are damaged and their content is released into the bloodstream. [7] Detection of dd-cfDNA is based on chimerism: donor cells are genetically distinct from that of the transplant recipient [6].

Immunological analysis of the kidney explant showed marked graft infiltration with alloreactive PD-1^+^ cytotoxic T cells that were saturated with nivolumab, demonstrating nivolumab-mediated inhibition of PD-1. This indicated that nivolumab was bound to the T cells which likely caused allograft rejection. The graft infiltrating T cell population had the capacity to mount an effector response.

As indications of ICIs are expected to expand and SOT recipients have an increased risk to develop malignancies, e.g. advanced hepatocellular carcinoma in liver transplant patients, the use of ICIs in SOT recipients is a clinical problem, the magnitude of which is likely to increase in the near future [11]. However, clinical trials of ICIs excluded SOT patients. Apart from case reports and case series [12–16], the efficacy and toxicity of ICI in transplanted patients with malignancies have not been studied extensively but do indicate the high risk of allograft rejection. Serum creatinine, which estimates the glomerular filtration rate, is not specific nor sensitive for kidney transplant rejection [17].

The findings of the present case study suggest that dd-cfDNA may be a valuable biomarker for early detection of ICI-induced transplant rejection. It remains unclear at this stage if this novel biomarker outperforms conventional biomarkers such as serum creatinine. The first serum creatinine measurement in this case was only performed 12 days after the first administration of nivolumab and not at the same time of the dd-cfDNA measurement.

In conclusion, physicians prescribing ICIs should be aware of the increased risk of allograft rejection as a result of T cell activation. We believe that a combined measurement of dd-cfDNA and conventional biomarkers may assist physicians to diagnose transplant rejection in this particular setting at an early stage but this should be studied prospectively. The transplant rejection was caused by alloreactive cytotoxic T cells that were positive for PD-1 and were saturated with nivolumab, which is in line with the anti-tumor effect of this drug.

## Methods

### Genotyping, isolation and measurement of dd-cfDNA

Peripheral blood mononuclear cells of the recipient and spleen cells of the donor were used for automated purification of DNA (Maxwell, Promega, Leiden, the Netherlands). Donor and recipient were genotyped and discriminated by using a panel of 10 preselected different single-nucleotide polymorphisms (SNP). Blood samples for dd-cfDNA were collected in CellSave BCT tubes (Menarini, San Diego, CA). Blood collection tubes were stored at 4 °C within 3 h after collection, and within 2 days post draw, plasma was separated by centrifugation at 1600×*g* for 20 min and stored at − 80 °C. Post thaw, plasma was centrifuged for a second time at 16,000×*g* for 10 min and cfDNA was extracted immediately using the Circulating Nucleic Acid kit (Qiagen, Venlo, The Netherlands)). For the droplet digital PCR (ddPCR), droplets were manually generated with the QX200 Droplet Generator (Bio-Rad, Lunteren, The Netherlands). The samples were run on a the T100™ Thermal Cycler (Biorad, Lunteren, The Netherlands). Dd-cfDNA was quantified based on differences in SNPs between donor and recipient (3 different SNPs that were able to distinguish between ddcfDNA and cfDNA) using the QX200™ Droplet Reader (Biorad, Lunteren, The Netherlands). Analysis was performed with QuantaSoft Analysis Pro (Bio-Rad, Lunteren, The Netherlands).

### Immunohistochemical stainings

Four μm sections of Formalin-Fixed Paraffin-Embedded (FFPE) tissue were mounted serially on adhesive glass slides and deparaffinized. Antigen retrieval was performed by CC1 antigen retrieval solution (ref. 950–124, Ventana Medical Systems, Inc., Oro Valley, Arizona). Specimens were incubated with the primary antibody. The following antibodies were used; CD3 (ref. 790–4341, Ventana Medical Systems, Inc., Oro Valley, Arizona), CD4 (ref. 790–4423, Ventana Medical Systems, Inc., Oro Valley, Arizona), CD8 (ref. 790–4460, Ventana Medical Systems), CD20 (790–2531 Ventana Medical Systems), Granzyme B (262R-18, Cell Marque Corporation, Rocklin, California), Ki-67 (ref. 790–4286 Ventana Medical Systems) and PD-1 (ref. 760–4895, Cell Marque). Detection was performed with OptiView DAB (ref. 760–700, Ventana Medical Systems, Inc.) or UltraView-DAB (ref. 760–500, Ventana Medical Systems, Inc) and amplification was done with the Amplification Kit (ref: 760–080 or OptiView Amplification Kit ref.: 760–099, Ventana Medical Systems, Inc.). Next, the specimens were counterstained with haematoxylin II (ref: 790–2208, Ventana Medical Systems, Inc.) and cover-slipped in order to keep the specimens pressed flat. Each slide contained a positive control. All stainings were performed on the VENTANA BenchMark ULTRA (Ventana Medical Systems, Inc.).

### Flow cytometric phenotyping of graft infiltrating lymphocytes (GILs)

GILs were stained with the following monoclonal antibodies (MoAb) in order to determine their phenotype: CD3, CD4, CD8, and PD-1. In order to measure the capacity of the cells to produce pro-inflammatory cytokines, the GILs were stimulated for 4 h with 0.5 μg/mL phorbol myristate acetate (PMA) and 10 μg/mL ionomycin (Sigma-Aldrich, St. Louis, MO) at 37 °C. Intracellular accumulation of cytokines was enhanced by the addition of monensin and brefeldin A. The reaction was stopped by the addition of ethylene-diamine-tetra-acetic acid. Subsequently, cells were stained with CD3 brilliant violet 510 (BV510; Biolegend, San Diego, CA), CD4 brilliant violet 421 (BV421; Biolegend), CD8 phycoerythrin-cyanine7 (Pe-Cy7; BD), PD-1 allophycocyanin-Cy7 (APC-Cy7; Biolegend), and the viability marker 7-aminoactinomycin (7-AAD; Biolegend). After surface staining, the cells were immediately fixed with FACS lysing solution (BD) and permeabilized with PERM II (BD). Intracellular staining was performed with the following MoAb: TNFα PE (Biolegend), IFNγ fluorescein isothiocyanate (FITC; BD) and IL-2 APC (BD). Samples were measured on the FACSCanto II (BD).

In order to determine free binding places of nivolumab (Bristol-Myers Squibb, New York, NY), was labelled with the SiteClick™ R-PE Antibody Labeling Kit (ThermoFisher, Waltham, MA). The GILs from the patient and the control patient were not stimulated. Cells were phenotyped with the following monoclonal antibodies: CD3 brilliant violet 510 (Biolegend), CD4 brilliant violet 421 (Biolegend), CD8 phycoerythrin-cyanine7 (BD), Nivolumab-PE, and the viability marker 7-aminoactinomycin (Biolegend). After surface staining, the cells were measured on the FACSCanto II (BD). Analysis was performed with Kaluza 1.5a software (Beckman Coulter, Brea, CA).

## Data Availability

The datasets used and/or analyzed during the current study are available from the corresponding author on reasonable request.

## Abbreviations

dd-cfDNA: Donor-derived cell-free DNA
GIL: Graft infiltrating lymphocyte
ICI: Immune checkpoint inhibitor
SOT: Solid organ transplantation
